# Sexual dichromatism in the fur of a bat: An exploration of color differences and potential signaling functions

**DOI:** 10.1002/ece3.11023

**Published:** 2024-02-16

**Authors:** Elizabeth A. Beilke, Jahshua F. Sanchez, Diana K. Hews, Joy M. O'Keefe

**Affiliations:** ^1^ Department of Natural Resources and Environmental Sciences University of Illinois at Urbana‐Champaign Urbana Illinois USA; ^2^ Department of Biology Indiana State University Terre Haute Indiana USA

**Keywords:** Chiroptera (bats), fur color, *Lasiurus borealis*, pelage, sexual dichromatism, sexual dimorphism

## Abstract

Sex differences in body color (i.e., sexual dichromatism) are rare in bats and, more broadly, in mammals. The eastern red bat (*Lasiurus borealis*) is a common tree‐roosting bat that occupies much of North America and has long been described as sexually dichromatic. However, previous research on this species found that absolute body size and collection year were better predictors of fur color in preserved specimens than sex. We revisited this issue and photographed 82 live eastern red bats under standardized conditions, then used image analysis to quantify pelage hue, saturation, and value. We used an information theoretic approach to evaluate four competing hypotheses about the principal drivers of color differences in the fur of eastern red bats. Our analyses demonstrated that sex was a better predictor of pelage color than body size; males had redder, more saturated, and lighter pelages than females. Additionally, the fur color of juvenile versus adult bats differed somewhat, as juveniles were darker than adults. In general, absolute body size (i.e., forearm length in bats) was a poor predictor of color in live eastern red bats. In an exploratory post‐hoc analysis, we confirm that fur color is related to body mass (i.e., a proxy for body condition in bats), suggesting color might serve as a sexually selected signal of mate quality in this partially diurnal species. Future work should investigate the functional role of sexual dichromatism in this species, which could be related to signaling or possibly thermoregulation.

## Graphical abstract

We quantified the fur color of live eastern red bats (*Lasiurus borealis*) and demonstrated that they are sexually dichromatic; males had redder, more saturated, and lighter pelages than females. Additionally, juveniles were darker than adults. In an exploratory post‐hoc analysis, we showed that body mass (i.e., an index of body condition in bats) strongly predicted fur color, revealing the potential for color to be a sexual signal in this species.

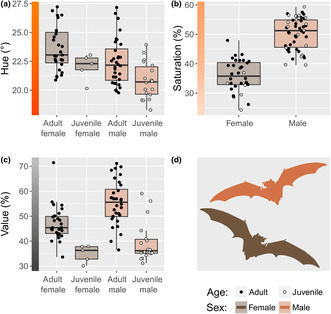

## 1 INTRODUCTION

Sexual dichromatism refers to the variation in coloration between the sexes of a species. The causes of sexual dichromatism are varied, and they have been studied from both proximate and ultimate perspectives. Proximally, sex differences in hormones in adults can impact the production or deposition of pigments that create colors in the fur, skin, feathers, or scales (Ducrest et al., 2008; Glucksmann, 1974; Hews & Moore, 1995; Lindsay et al., 2011), and may also require early developmental exposure to sex steroids. Bright colors in many vertebrates (e.g., birds, fish, lizards) are achieved via three broad categories of mechanisms: carotenoid pigments acquired through foraging and incorporated into the integument, eumelanin and phaeomelanin pigments that are synthesized de novo, and structural colors arising from nanostructural arrangements (Hill & McGraw, 2006). Sexual dichromatism is uncommon in mammals despite their having dichromatic vision (Jacobs, 2009). Also, color in mammalian fur is achieved only by using eumelanin, phaeomelanin, or both (Caro & Mallarino, 2020), which has two main consequences. First, mammalian fur is less colorful than comparable structures in other animals. Second, because these pigments are made from molecules not acquired through foraging, the resulting colors may not be good indicators of body condition and, thus, may be less useful as sexual signals (see Badyaev & Hill, 2000).

Over evolutionary time, various forms of selection have led to sexual dichromatism in vertebrates (Tobias et al., 2012). For example, passerine birds that exhibit strong male‐biased sexual selection tend to have sexually dichromatic plumage with color‐elaborated males and cryptically colored females (Badyaev & Hill, 2003; Dale et al., 2015; Dunn et al., 2001). Patterns of sexual dichromatism may ultimately arise from various factors, including animal distributions in space and time, mating systems, parental care, pressures for crypsis, and how animals interact with habitat and prey (Badyaev & Hill, 2003); these factors may contribute to its evolution (Shine, 1989) or constrain it (e.g., Stuart‐Fox & Ord, 2004). In contrast to other animals, color may be less important for sexual signaling in mammals, even though mammals exhibit mate choice behaviors (Caro & Mallarino, 2020).

Sexual dichromatism may be rare in bats; only a few accounts of sexually dichromatic species exist. For instance, the pelages of male Waterhouse's leaf‐nosed bats (Phyllostomidae: *Macrotus waterhousii jamaicensis*), great fruit‐eating bats (Phyllostomidae: *Artibeus lituratus*), and Mexican long‐tongued bats (Phyllostomidae: *Choeronycteris mexicana*) have all been described as redder than those of their female counterparts (Goodwin, 1970; Lukens Jr. & Davis, 1957). Honduran white bats (Phyllostomidae: *Ectophylla alba*) also show sexual dichromatism: male Honduran white bats have noses that are a brighter shade of yellow than those of females (Rodríguez‐Herrera et al., 2019). It is possible that sexual dichromatism is not rare, but is instead poorly documented in bats, which are an understudied taxon (Frick et al., 2020). Over 150 years ago, Dobson (1873) noted of several bat species that “where male and female specimens of the same species [had] been obtained at the same place and time, the lighter‐coloured specimens [were] invariably males.”

Eastern red bats (Vespertilionidae: *Lasiurus borealis*) are common North American tree‐roosting bats that have long been described as sexually dichromatic (e.g., Cryan, 2008; Timm, 1989; Whitaker & Hamilton, 1998), but this description has yet to hold up to scientific scrutiny. Davis and Castleberry (2010) measured the pelage hue of dry eastern red bat specimens and reported that absolute body size (i.e., body length) was a stronger predictor of hue than sex. Thus, they concluded that the sexual dichromatism apparent in eastern red bats is instead an artifact of the relationship between sexual size dimorphism and color—smaller individuals, which are more likely to be male, are also more red (Davis & Castleberry, 2010). However, the authors observed a significant relationship between fur color and the age of the museum specimens, which were collected between 1940 and 1993 (i.e., specimens were roughly 15–70 years old when examined). In a follow‐up study, Davis et al. (2013) reported that a few species of reddish‐colored mammalian specimens grew redder the longer they were in storage, possibly due to the degradation of eumelanin pigments in the fur. Ultimately, they recommended that their study be repeated using live specimens to eliminate the confounding influence of specimen age on fur color measurements.

Following this recommendation, we used live bats to test the hypothesis that eastern red bats are sexually dichromatic and evaluated the possible effects of absolute body size and age on fur color. We captured eastern red bats, noted their sex, age, forearm length, and body mass, and photographed them under standardized conditions to quantify fur color using image analysis. We predicted that if eastern red bats are sexually dichromatic, sex would better predict fur color than absolute body size (i.e., forearm length). We also performed an exploratory post‐hoc analysis to test the hypothesis that fur color is a sexual signal of mate quality. If true, color should relate to an index of mate quality, such as body condition (i.e., fat mass; McGuire et al., 2018) during the time of the moult, which is thought to occur in summer (Fraser et al., 2013) prior to the onset of fall breeding. Body condition during the summer moult could thus be reflected in the fur and relevant for mate choice.

## 2 METHODS

### 2.1 Study species

Eastern red bats are forest‐dependent bats broadly distributed across much of North America. They roost in the foliage of tree canopies (Shump & Shump, 1982), either alone or in small family groups. Eastern red bats produce broadband, moderate‐frequency echolocation calls and have high wing loading scores, which makes them well suited for flying and capturing insects in semi‐open to open spaces (Aldridge & Rautenbach, 1987; Norberg & Rayner, 1987). They forage in various forest habitats (Elmore et al., 2005), often focusing their foraging behavior along linear features within the forest matrix, such as ridgetops and roads (Amelon et al., 2014; Beilke et al., 2023). They consume a wide variety of insects, though moths and beetles may be favored prey items (Clare et al., 2009; Hayes et al., 2019; Whitaker, 2004). The species engages in partial seasonal migration, wherein some individuals travel north in the spring and south in the fall (Cryan, 2003; True et al., 2023; Wieringa et al., 2021). Mating occurs in autumn, presumably during migration, at which point young‐of‐year juveniles have reached sexual maturity (Cryan et al., 2012).

**FIGURE 1:**
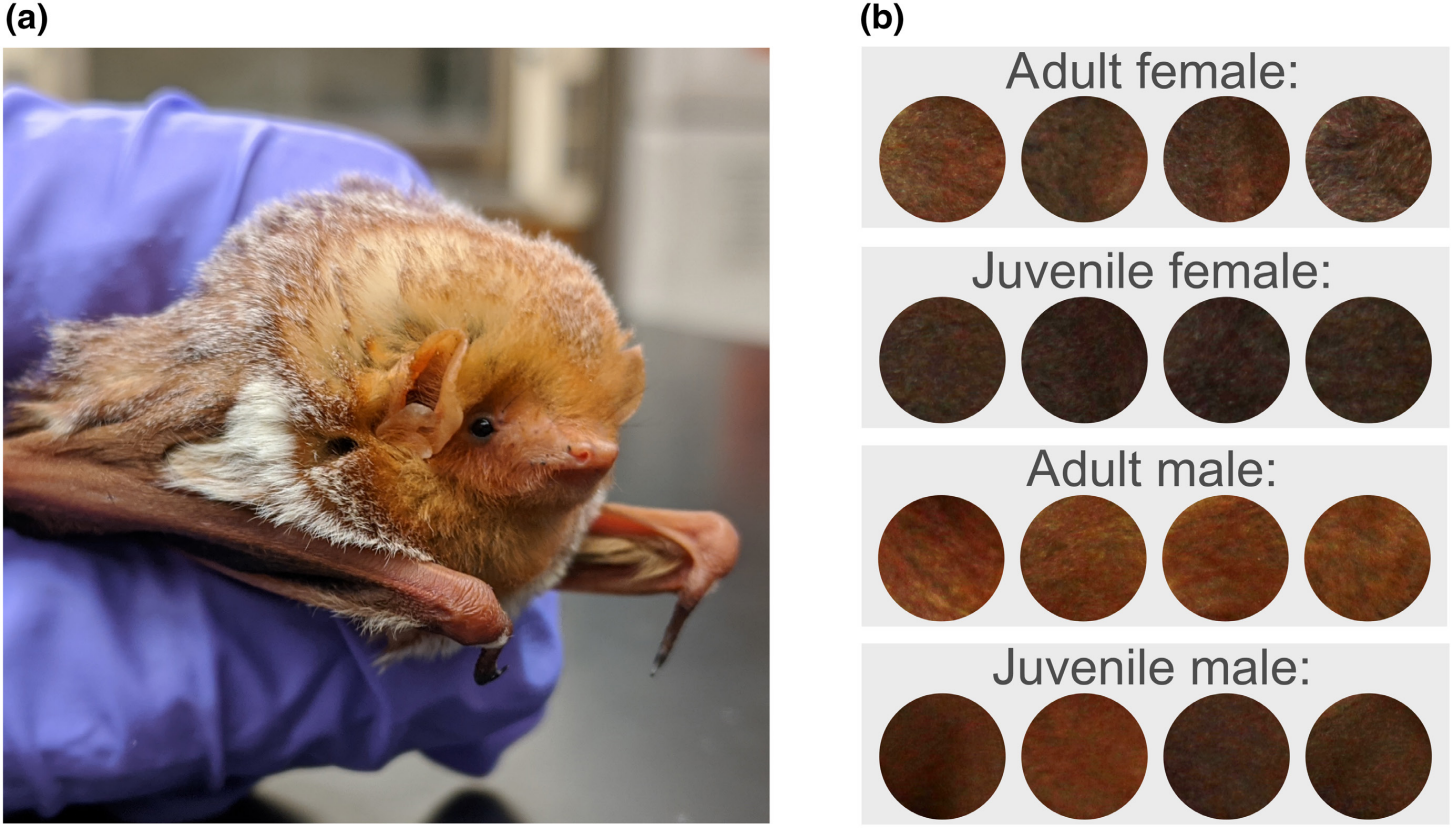
An adult female eastern red bat (*Lasiurus borealis*) under well‐lit conditions (a) and examples of the pelage variation observed within the species, based on sex and age (b). All images used in panel (b) are clipped from random study photos, which were taken under standardized conditions. All photos were taken by the authors.

Eastern red bats have an ornate pelage (Figure 1), consisting of a thick coat of bright fur covering most of their body, including their tail and parts of the wing membrane. Each hair strand has banded coloration (i.e., agouti): dark at the base, transitioning from yellow to orange or brown in the middle, and can be white at the tip. In addition to being brightly colored, eastern red bats exhibit a variety of pelage markings that may be related to disruptive camouflage (see Santana et al., 2011), including counter‐shading, a white neck bar, and small white patches near their first digits and shoulder blades. The species is also size dimorphic, with females being the larger sex (Williams & Findley, 1979).

### 2.2 Study area

We conducted this study in Morgan‐Monroe (39.3° N, 86.4° W) and Yellowwood State Forests (39.1° N, 86.3° W), two contiguous hardwood forests in Morgan, Monroe, and Brown counties in south‐central Indiana, USA. These state forests span 20,000 contiguous ha and are managed by the Indiana Department of Natural Resources for multiple uses, including recreation, timber production, and conservation. Elevation ranges from 490 to 1057 m above sea level, and the landscape is characterized by ridgetops, steep ravines, and drains with streambeds that hold water intermittently throughout the summer. Eastern red bats are one of the area's most commonly captured bat species (Beilke & O'Keefe, 2022).

### 2.3 Data collection

#### 2.3.1 Bat capture

We captured eastern red bats at 16 sites across the study area from May 20 to August 13, 2021; this is the typical survey season for bats in the region. We selected sites on or near potential bat travel corridors or drinking sources, including streams, upland forest corridors, bottomland forest corridors, and ponds. Sites ranged from 640 m to 29 km apart (mean minimum distance between sites: 2114 m). To capture bats, we deployed 2–6 mist nets per site. We used a variety of single, double, and triple‐high mist nets, which ranged from 6 to 18 m in length (Avinet, Portland, ME, USA). We opened mist nets at or just after dusk, netting for 1–4 h; we sometimes closed early for inclement weather.

We recorded the sex, reproductive condition, forearm length, body mass, and age (juvenile or adult) of each individual. We measured forearm length using DialMax calipers (0.1 mm resolution; Wiha, Burlington, ON, Canada) and body mass using a Pocket Pro digital scale (0.1 g resolution; Ohaus, Parsippany, NJ, USA). We determined age by examining the degree of ossification in the metacarpal‐phalangeal joints, which are not fully ossified in young‐of‐year bats (Brunet‐Rossinni & Wilkinson, 2009). We began capturing lactating eastern red bats around June 14, and the last juvenile we photographed was captured on August 13. Thus, all juvenile bats we photographed were likely <2 months old. We did not apply unique marks to bats. However, we trimmed fur from the backs of each captured individual (after photography, see below) to allow identification of recaptures within a night or across consecutive net nights. We surveyed several sites multiple times throughout the summer but only photographed bats again at the same site when they were clearly unsampled individuals (representing a different sex or age group). Our final sample size included 82 individual bats: 50 males (32 adults and 18 juveniles) and 32 females (27 adults and five juveniles).

We followed the guidelines of the American Society of Mammalogists for the use of wild mammals in research (Sikes et al., 2019), institutional animal care and use protocols (University of Illinois Urbana‐Champaign, #21022), and all white‐nose syndrome decontamination recommendations (White‐nose Syndrome Disease Management Working Group, 2020). We held appropriate Indiana (3195) and USFWS permits (ES206872).

#### 2.3.2 Photography

We performed all photography in the field. We held each bat in a standardized position (wings closed, forearms held against body and uropatagium spread, with bats' ventral surface down) and photographed them in a portable LED‐lit photo studio box (33 × 36 × 41 cm box containing 52 10 W, 5500 K LED lights; EMART International Inc., Westminster, CA, USA) using a digital single‐lens reflex camera (EOS Rebel T6) with an EFS 18–55 mm lens (Canon, Tokyo, Japan). To achieve homogeneous lighting conditions, we did not use flash to photograph bats but relied only on the illumination produced by the two LED strips within the photo studio box. We photographed the bats from a standardized distance (36 cm) with camera settings at IOS 800, a shutter speed of 1/800th of a second, and f/5.6 (10.9 mm). We photographed bats against a neutral gray background and included a color correction card within each photograph. We photographed and stored all images in large format, a setting that optimizes photo quality but is not memory intensive (typical file size: 5700 KB).

#### 2.3.3 Color analyses

We performed all color analyses in ImageJ version 1.8.0 (Rasband, 2018; Schneider et al., 2012). For this study, we used hue, saturation, and value scores to describe fur color. Hue describes colors in their purest forms (e.g., red, blue, etc.). Hue scores range from 0 to 359°, with shades of red, orange, and yellow occurring from 0 to 30°. Saturation describes the purity or intensity of any color, expressed as a percent. Lower saturation scores indicate less vibrant colors. Value describes how close a color is to white or black (i.e., brightness) and is also expressed as a percent. Lower value scores indicate darker colors. To quantify the color of bat fur, we selected the furred dorsal surface of each bat's exposed uropatagium (the membrane that extends between the hind limbs) to measure (hereafter, “selected area”). For each bat, we quantified the average hue, saturation, and value scores across the selected area. We also quantified color across the whole of each bat's exposed dorsal surface. Ultimately, the color scores of the two surface types were highly correlated (Pearson's correlation coefficients >.90; data not shown), and we opted to use the uropatagium scores to facilitate comparisons with earlier work by Davis and Castleberry (2010). To assess whether variation in the size of the selected area influenced our analysis, we quantified the number of pixels within each selected area. Ultimately, the selected area size was not related to hue, saturation, or value scores (linear models, *F*_1,78_ = 0.80, 0.04, and 0.19; *p* = .373, .838, and .661, respectively), and we did not include it as a covariate in our models. Overall, these scoring techniques produced reliable measurements that could be consistently replicated (for details on reliability analyses, see Appendix S1).

We also quantified the exposure of each image by measuring the mean brightness of a medium‐light shade of gray from the color correction card we included in each image. In ImageJ, brightness is calculated as the average of red, green, and blue scores (values range from 0 to 255, i.e., black to white). Brightness scores were highly consistent across all images (i.e., no outliers; 174 ± 8, mean ± SD). Thus, we concluded that any color differences we observed among bats were not artifacts of lighting or exposure.

### 2.4 Statistical analysis

All response variables were normally distributed. Therefore, we used linear models to examine the relationship between response and predictor values. Our candidate model set included four models for each response variable (hue, saturation, and value): three models testing different a priori hypotheses and a null model (see Table 1). We used an information‐theoretic approach to compare models (Burnham & Anderson, 2002). To identify the most plausible model within our candidate model set, we required models to have substantial *support* (∆AICc ≤ 2.0; “confidence set”). When multiple models were in our confidence set, we evaluated the most parsimonious model. Within these models, we considered parameters important if the 85% confidence interval values of the parameter estimate did not cross zero (Arnold, 2010). We assessed the fit of the models in the confidence set by checking their residual plots. Model residuals were normally distributed and exhibited no clear patterns (i.e., heteroskedasticity).

**TABLE 1:** An overview of the candidate models tested for each response variable (hue, saturation, and value), including the variables in each model and a description of the hypothesis the model tested.

Model name	Variables included	Hypothesis summary
Sex	Sex (male versus female)	Sexual dichromatism; fur color will vary with sex. Based on the observation that male and female eastern red bats display different pelage coloration (e.g., Whitaker & Hamilton, 1998)
Sex × Age	Sex (male versus female) × age (adult versus juvenile)	Sexual dichromatism plus age‐dependent coloration; color will vary with sex and/or age. Based on the observation that male and female eastern red bats display different pelage coloration and that juvenile bats are darker than adults (e.g., Timm, 1989)
Body size	Forearm length	Size‐dependent coloration; fur color will vary with absolute body size. Based on the observation that absolute body size is a stronger predictor of pelage color than other variables, including sex (e.g., Davis & Castleberry, 2010)
Null	1	Fur color does not vary with sex, age, or absolute body size

In an exploratory post‐hoc analysis, we tested the potential for fur color to be a sexual signal, possibly of mate quality. We examined the relationship between body mass and saturation, which had the most robust relationship with sex in our initial analysis. We used body mass as a proxy for body condition, as body mass strongly correlates with fat mass in bats and outperforms other common body condition indices (McGuire et al., 2018). For this analysis, our candidate model set included three linear models: one relating saturation to body mass while accounting for sexual dichromatism (saturation ~ mass + sex), one to examine the possibility that saturation has a sex‐specific relationship with body mass (saturation ~ mass × sex), and a null model. We excluded pregnant females from this analysis. We identified the most plausible model and interpreted parameter importance using the abovementioned methods.

We performed all analyses in R version 4.2.0 (R Core Team, 2022) and RStudio version 2022.02.3 (RStudio Team, 2022). We produced figures using the ggplot2 and patchwork packages and used Inkscape version 1.2 for post‐production (Inkscape Project, 2021; Pedersen, 2020; Wickham, 2016).

## 3 RESULTS

Our most complex model, which included sex, age, and the interaction between sex and age, was the most plausible model for all three response variables (hue, saturation, and value; Table S1 in Appendix S1). However, a more parsimonious model, containing only sex as a predictor variable, was also in the confidence set for saturation (Table S1 in Appendix S1), revealing that the interactive effect between sex and age did not significantly improve our top model. The forearm length model did not appear in any confidence sets and, thus, forearm length was deemed a poor predictor of fur color (Figure S1 in Appendix S1). All models outperformed the null model in their respective candidate model sets.

**FIGURE 2:**
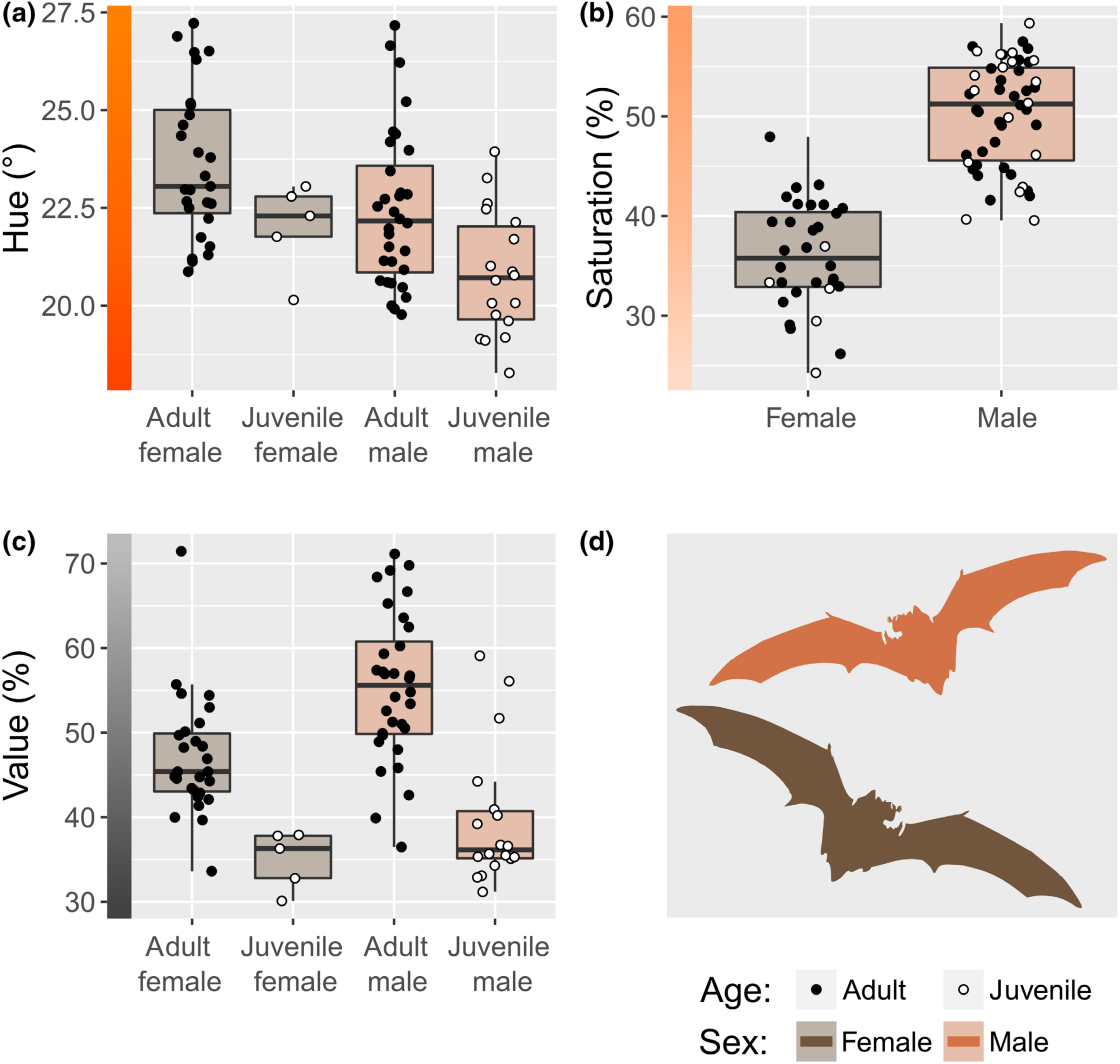
Eastern red bats (*Lasiurus borealis*, *n* = 82) are sexually dichromatic in the hue (a), saturation (b), and value (c) of their fur color (d). Additionally, juvenile males exhibit lower hue scores than adult males (a), and juvenile bats (open circles) exhibit lower value scores than adult bats (closed circles; c). Colored bars on the vertical axes are visual aids corresponding to raw hue, saturation, and value scores. It is impossible to visualize saturation without hue, and thus we held hue at a mean value (23) to produce the colored bar for panel (b). Panel (d) shows visual representations of fur color for male (orange) and female (brown) bats based on the distal scores presented in panels (a–c).

Sex and age strongly predicted hue scores in eastern red bat fur. Hues ranged from reddish (18°) to orange (27°; Figure 2a). Males were redder in hue than females (β = −1.17, CI = −1.88 to ‐0.47; Figure 2a, Table 2), and juveniles were redder in hue than adults (β = −1.64, CI = −2.95 to ‐0.33, Figure 2a, Table 2). On average, males were 5% closer to pure red (i.e., hue 0°) than females, and juveniles were 4–10% closer to pure red than adults of the same sex.

**TABLE 2:** Output for the most parsimonious and plausible models relating fur color variables (hue, saturation, and value) to demographic variables (sex and age) for eastern red bats (*Lasiurus borealis*).

Color variable	Covariate	Estimate	Standard error	85% confidence interval	
				Lower	Upper
Hue	Intercept: Adult Female	23.58	0.36	23.06	24.09
	Sex: Male	−1.17	0.48	−1.88	−0.47
	Age: Juvenile	−1.64	0.90	−2.95	−0.33
	Sex: Male × Age: Juvenile	−0.00	1.05	−1.53	1.53
Saturation	Intercept: Female	35.93	0.95	34.54	37.31
	Sex: Male	14.34	1.22	12.57	16.11
Value	Intercept: Adult Female	47.06	1.52	44.85	49.26
	Sex: Male	8.33	2.06	5.33	11.32
	Age: Juvenile	−12.08	3.84	−17.66	−6.49
	Sex: Male × Age: Juvenile	−3.69	4.49	−10.21	2.83

*Note*: Output includes model estimates, standard errors, and 85% confidence intervals.

Sex was the only strong predictor of saturation scores in eastern red bats. Saturation (or purity of color) scores ranged from 24% to 59% (Figure 2b). On average, male fur was 40% more saturated than female fur, meaning they were a purer or more concentrated shade of red than females (β = 14.34, CI = 12.57 to 16.11; Figure 2b, Table 2). Age was not a strong predictor of saturation scores. Thus, juvenile eastern red bats already exhibited some pelage characteristics of their sexually dichromatic adult counterparts.

Sex and age predicted value scores in eastern red bat fur. Value (or brightness) scores ranged from 30% to 71% (Figure 2c). Female fur was 15% darker than male fur (β = 8.33, CI = 5.33 to 11.32; Figure 2c, Table 2), and juvenile bats were darker still (β = ‐12.08, CI = −17.66 to ‐6.49; Figure 2c, Table 2). On average, juvenile fur was approximately 26–31% darker than the fur of adult bats of the same sex.

**FIGURE 3:**
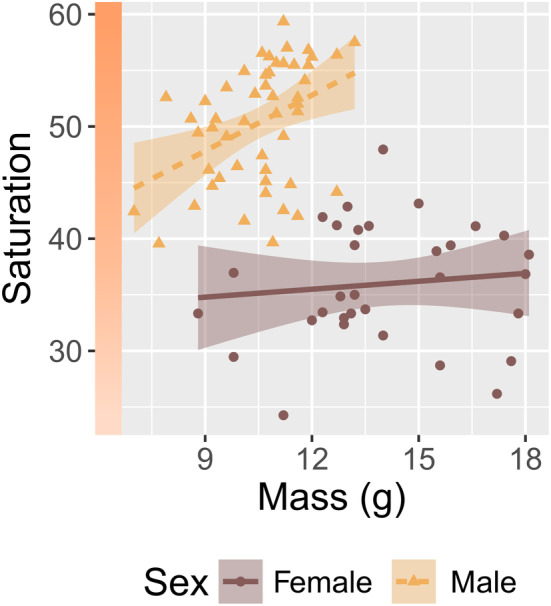
Body mass (i.e., body condition; McGuire et al., 2018) is related to fur color saturation in both male (triangles, dashed line; *n* = 50) and female (dots, solid line; *n* = 22) eastern red bats. We excluded pregnant females from this analysis. On the plot, data points represent raw scores, shading denotes 95% confidence intervals surrounding linear model output, and the colored bar on the vertical axis is a visual aid corresponding to raw saturation scores while holding hue at a mean value (23°).

The top model in our post‐hoc analysis included body mass and the additive effect of sex, revealing that body mass (i.e., a proxy for body condition) was a strong predictor of pelage saturation scores in eastern red bats (β = 1.51, CI = 0.93 to 2.08). In both sexes, body mass was positively associated with saturation (Figure 3).

## 4 DISCUSSION

Here, we provide evidence for sexual dichromatism in eastern red bats, with males exhibiting more vibrant fur color than females. We also report that relative body size (a proxy for body condition) was positively associated with fur color in both male and female bats. This suggests color could function as a sexual signal in this species, though other ultimate factors could mediate the differences in fur color we observed. Additionally, we find that sexual dichromatism is already apparent in young‐of‐year juvenile bats, though they are darker overall when compared to adults. Our study is one of only a few to document the occurrence of sexual dichromatism in bat fur (see also Dobson, 1873; Goodwin, 1970; Lukens Jr. & Davis, 1957) and challenges previous work on this species reporting that absolute body size is a better predictor of fur color than sex (Davis & Castleberry, 2010). These observations could reveal new insights into the behavior of eastern red bats and, more broadly, the ecology and evolution of sexual dichromatism in bats and other mammals.

Androgens may mediate some of these differences in fur color in eastern red bats via effects on melanin, and this likely proximate mechanism has implications for functional hypotheses. The range of melanin‐based colors we documented likely resulted from different combinations of eumelanin and phaeomelanin (Prota et al., 1995). Male vertebrates usually have higher androgens than females (Lindsay et al., 2016; Staub & De Beer, 1997), and elevated plasma androgens—and perhaps phaeomelanin (e.g., Lindsay et al., 2016)—typically stimulate higher eumelanin content in feathers and fur (Haase et al., 1995; Slominski et al., 2004). This could explain why male eastern red bats exhibit a richer red hue than females. Colorations involving melanin and, hence, the melanocortin system are complex as this system has multiple functions—regulating sebaceous glands, antibiotic activity, resistance to UV radiation and oxidative stress, food intake, reward pathways, blood pressure, glucose control, and energy expenditure. Hence, the red in eastern red bats could be evidence of a suite of traits related to androgen production (Ducrest et al., 2008) and could be tied to sexual selection. However, specific functional hypotheses for sexual dichromatism involving androgens and the melanocortin system await a greater understanding of these traits in eastern red bats.

Body mass, a proxy for body condition in bats (McGuire et al., 2018), was positively associated with fur color saturation in male and female eastern red bats. Given that many sexual signals are related to body condition in vertebrates, this relationship suggests color could function as a sexual signal in eastern red bats. Sexual dichromatism in eastern red bats may have evolved due to sexual selection (Dale et al., 2015), possibly to convey information about individual fitness. This hypothesis is supported by evidence that young‐of‐year juveniles are already sexually dichromatic by the time of sexual maturity (i.e., in hue and saturation) despite retaining some characteristics unique to juvenile pelages (i.e., dark fur). The color differences between male and female eastern red bats could also reflect a balance between competing selection pressures for color elaboration in reproductive males and crypsis in non‐reproductive juveniles and females (as is the case for many sexually dichromatic passerine birds; Dale et al., 2015). Eastern red bats exhibit pelage markings that suggest natural selection has favored crypsis in this species (e.g., countershading, a white neck bar, and small white patches near their first digits and shoulder blades; see Santana et al., 2011). While natural selection may generally favor crypsis, we suggest that bright orange fur occurs in male eastern red bats due to sexual selection for color as a signal of mate quality. However, we caution that we do not know what colors are associated with crypsis in this species. Male and female pelages may both be cryptic in their respective roosting conditions, given that the sexes vary in their roosting behaviors (Beilke et al., 2023; Elmore et al., 2004).

For fur color to be a sexual signal, it must first be perceived. Assuming eastern red bats can discern color differences in conspecific pelages, this would require signal discrimination to occur in lit conditions, as light is essential to the perception of color. This is a limitation of the hypothesis that fur color is a sexual signal among eastern red bats, as the species is mainly thought to be nocturnal. However, these bats sometimes fly in well‐lit conditions, where conspecifics could detect fur color. Eastern red bats are frequently observed flying during the daytime, as supported both by community science databases (e.g., iNaturalist; GBIF.org, 2022) and technical publications (Barbour & Davis, 1969; Hatch et al., 2013). Furthermore, mating likely coincides with migration (Glass, 1966; Stuewer, 1948), and eastern red bats have been observed migrating during the day (Barbour & Davis, 1969; Hatch et al., 2013). Eastern red bats also roost in well‐lit conditions (i.e., in tree canopies), can emerge to forage before sunset (Beilke et al., 2023), exhibit peaks in activity at dawn and dusk (Beilke et al., 2021), and do not avoid moonlight (Arndt, 2018) or artificial light at night (Seewagen & Adams, 2021). Thus, mating events may frequently occur while there is enough ambient light for bats to discern fur color. If color is a sexual signal, it would be important to explore how ambient lighting varies with habitat (e.g., forest versus open fields), as color perception would be affected (Endler, 1993). We don't know much about bat vision, though there is an increasing body of evidence to suggest many bats possess dichromatic color vision and sometimes ultraviolet vision (e.g., Simões et al., 2019; Wang et al., 2004; Zhao et al., 2009). Even if bats cannot perceive differences in the hue of color, it is still possible these bats can discern the differences between other aspects of color (e.g., value; darkness).

Sexual dichromatism does not necessitate sexual selection and may be an adaptive trait that has evolved through sexual niche partitioning (e.g., Heinsohn et al., 2005). Among bats, there are significant sex‐based differences in thermal and energetic requirements, such that males and females often have divergent roosting habits (Perry et al., 2007) and use torpor differently (Speakman & Thomas, 2003). Sexual dichromatism in eastern red bats could be related to thermoregulation, as the amount of light absorbed by fur depends on its color. Pregnant, lactating, and juvenile bats use less daily torpor than males or nonreproductive females, as torpor inhibits milk production and depresses pup development (e.g., *Myotis daubentonii*; Dietz & Kalko, 2006). As such, reproductive female bats often select roosts with warmer microclimates, which help reduce the energy costs of defending normothermic body temperatures (Hamilton & Barclay, 1994; Klug et al., 2012). Thus, they may benefit from a darker pelage that absorbs more incident solar radiation. Conversely, adult male bats use torpor more often and more deeply (Dietz & Kalko, 2006; Hamilton & Barclay, 1994). Males are less selective of roost microclimate (Alston et al., 2022) and could benefit from a lighter pelage that absorbs less solar radiation, allowing them to enter torpor more readily.

This work has a few limitations, which bear mentioning. First, it is very difficult to estimate the age of adult bats accurately. Thus, it is a common practice to classify the age of bats as either young‐of‐year juveniles or adults. However, it is probable that the fur color of bats undergoes substantial changes throughout the course of their long lives (Caro & Mallarino, 2020). To fully understand the physiology and ecology of bats, we must develop better techniques to accurately assess the age of these animals in the field (Wilkinson et al., 2021). Another key limitation of this work is that we photographed these animals during a relatively brief time period—three months. Though most bats are thought to moult only once a year (during the summer before migration and mating; Fraser et al., 2013), it is possible that there is some seasonal component to fur coloration in this species. Similarly, we examined fur color over a narrow geographic range. Future work might consider examining the possibility of range‐wide variation in the pelage color of this species (a possibility noted by Whitaker & Hamilton, 1998); fur color might vary with climate or available roosting habitat, for example.

Here, we show that sex strongly predicts fur color in eastern red bats. We also demonstrate that fur color is correlated with body condition, suggesting fur color could act as a sexual signal in this species. Future work should focus on documenting the functional roles of sexual dichromatism in eastern red bats, which will likely require studying their reproductive behaviors in greater detail. Other bat species should be examined for sexual dichromatism, including close relatives of the eastern red bat (e.g., *L. seminolus* and *L. blossevilli*; Baird et al., 2015). Understanding the roles and prevalence of sexual dichromatism in bats may help increase our understanding of the relative importance of vision in bats, as a growing body of evidence suggests that bats rely on visual cues more frequently than previously thought (Corcoran et al., 2021; Danilovich & Yovel, 2019; Gorresen et al., 2015; Jones et al., 2013). Bats could serve as model organisms for studying the evolution of sexual dichromatism, particularly within mainly nocturnal animals.

## AUTHOR CONTRIBUTIONS

**Elizabeth A. Beilke:** Conceptualization (lead); formal analysis (lead); investigation (equal); methodology (equal); project administration (equal); supervision (lead); validation (lead); visualization (lead); writing – original draft (lead); writing – review and editing (equal). **Jahshua F. Sanchez:** Investigation (equal); resources (lead); writing – original draft (supporting); writing – review and editing (equal). **Diana K. Hews:** Methodology (equal); writing – original draft (supporting); writing – review and editing (equal). **Joy M. O'Keefe:** Funding acquisition (lead); project administration (equal); writing – review and editing (equal).

## CONFLICT OF INTEREST STATEMENT

None declared.

## Supporting information


Appendix S1:

## Data Availability

The data that support the findings of this study are openly available in Dryad at https://doi.org/10.5061/dryad.37pvmcvr9.
