# Supplementary material for: Sexual dichromatism in the fur of a bat: An exploration of color differences and potential signaling functions
Source: Ecol Evol. 2024 Feb 16;14(2):e11023. doi: 10.1002/ece3.11023 (PMC10870327; doi:10.1002/ece3.11023)
Supplement: Supplementary file 1 — Appendix S1: [file ECE3-14-e11023-s001.docx]

Appendix S1. Supporting information for “Sexual dichromatism in the fur of a bat: an exploration of color differences and potential signaling functions.”

**File includes:**

Supplementary Text

Reliability Analyses

Supplementary Tables and Figures

Table S1

Figure S1

Supplementary Citations

**Authors:** Elizabeth A. Beilke^1*^, Jahshua F. Sanchez^1^, Diana K. Hews^2^, Joy M. O’Keefe^1^

^1^ Department of Natural Resources and Environmental Sciences, University of Illinois at Urbana-Champaign, W-503 Turner Hall, 1102 S. Goodwin, Urbana, Illinois, 61801, USA.

^2^ Department of Biology, Indiana State University. S-346, 600 North Chestnut Street, Terre Haute, IN 47809, USA.

* Corresponding author: Elizabeth Beilke ([lizz.beilke@gmail.com](mailto:lizz.beilke@gmail.com); [ebeilke@illinois.edu](mailto:ebeilke@illinois.edu))

# Supplementary Text

## Reliability Analyses

To assess test-retest reliability or measurement error, we photographed a subset of individual bats (n = 5) three times each, resetting the bat’s position in hand and the photo studio box each time. For these images, we quantified color as described above and calculated how strongly measures for each individual resembled each other (Yen and Lo 2002). This test was performed to examine the reliability of our holding techniques, as variation could be introduced due to a bat’s position in hand (e.g., the way parts of the body might be covered or the variation in the positioning of hair strands). We also selected a subset of photographs (n = 5) and quantified color as described above three times for each image, resetting ImageJ each time. We then tested how strongly photographs for each individual resembled each other. This test was performed to examine the reliability of our scoring techniques, as the polygon selection process could introduce variation. Reliability was high in both cases (intraclass correlation coefficient > 0.99). Intraclass correlation estimates were calculated using the irr package (Gamer et al. 2019) in R version 4.2.0 (R Core Team 2020) and RStudio version 2022.02.3 (RStudio Team 2022) and were based on a mean-rating, absolute-agreement, 2-way mixed-effects model (Koo and Li 2016).

# Supplementary Tables and Figures

**Table S1.** Models relating characteristics of eastern red bat (*Lasiurus borealis*) fur color (hue, saturation, and value), ranked by their Akaike Information Criterion values and model weights.

| Response Variable | Model | ΔAIC_c_ | ω_i_ |
| --- | --- | --- | --- |
| Hue | Sex * Age | 0.00 | 0.97 |
|  | Sex | 7.42 | 0.02 |
|  | Body Size | 14.40 | 0.00 |
|  | Null | 16.36 | 0.00 |
| Saturation | Sex * Age | 0.00 | 0.53 |
|  | Sex | 0.27 | 0.47 |
|  | Body Size | 54.02 | 0.00 |
|  | Null | 80.70 | 0.00 |
| Value | Sex * Age | 0.00 | 1.00 |
|  | Sex | 39.86 | 0.00 |
|  | Body Size | 40.53 | 0.00 |
|  | Null | 41.57 | 0.00 |

**
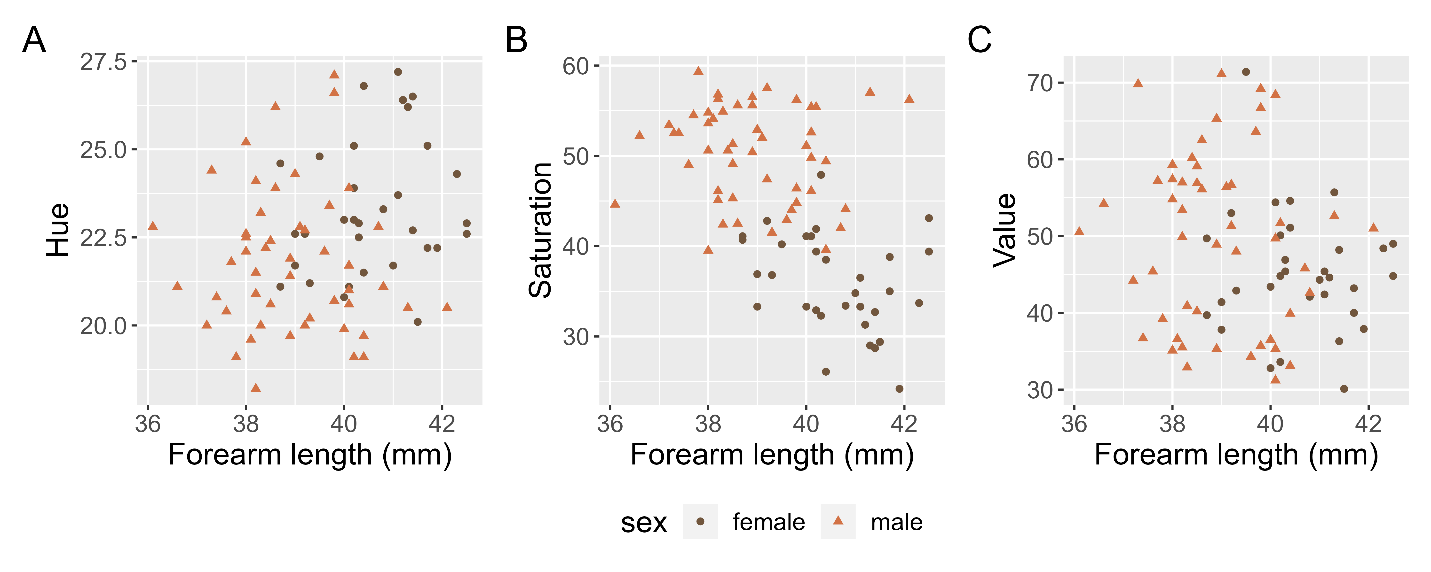
**

**Figure S1.** Absolute body size (i.e., forearm length) is a poor predictor of fur color hue (A), saturation (B), and value (C) in male (orange triangles; n = 50) and female (brown dots; n = 32) eastern red bats (*Lasiurus borealis*). On the plot, data points represent raw scores.

# Supplementary Citations

Gamer, M., J. Lemon, I. Fellows, and P. Singh. 2019. irr: various coefficients of interrater reliability and agreement.

Koo, T. K., and M. Y. Li. 2016. A guideline of selecting and reporting intraclass correlation coefficients for reliability research. Journal of Chiropractic Medicine 15:155–163.

R Core Team. 2020. R: A Language and Environment for Statistical Computing. R Foundation for Statistical Computing, Vienna, Austria.

RStudio Team. 2022. RStudio: Integrated Development Environment for R. RStudio, PBC, Boston, MA.

Yen, M., and L.-H. Lo. 2002. Examining test-retest reliability: an intra-class correlation approach. Nursing Research 51:59–62.
